# A method for analysing small samples of floral pollen for free and protein‐bound amino acids

**DOI:** 10.1111/2041-210X.12867

**Published:** 2017-10-16

**Authors:** Daniel Stabler, Eileen F. Power, Anne M. Borland, Jeremy D. Barnes, Geraldine A. Wright

**Affiliations:** ^1^ Institute of Neuroscience Henry Wellcome Building for Neuroecology Newcastle University Newcastle upon Tyne UK; ^2^ School of Natural Sciences Trinity College Dublin D2 Ireland; ^3^ School of Biology Newcastle University Newcastle upon Tyne UK

**Keywords:** acid hydrolysis, microwave hydrolysis, pollen amino acids, pollen nutrition, pollen wash, protein hydrolysis, UHPLC

## Abstract

1. Pollen provides floral visitors with essential nutrients including proteins, lipids, vitamins and minerals. As an important nutrient resource for pollinators, including honeybees and bumblebees, pollen quality is of growing interest in assessing available nutrition to foraging bees. To date, quantifying the protein‐bound amino acids in pollen has been difficult and methods rely on large amounts of pollen, typically more than 1 g. More usual is to estimate a crude protein value based on the nitrogen content of pollen, however, such methods provide no information on the distribution of essential and non‐essential amino acids constituting the proteins.
2. Here, we describe a method of microwave‐assisted acid hydrolysis using low amounts of pollen that allows exploration of amino acid composition, quantified using ultra high performance liquid chromatography (UHPLC), and a back calculation to estimate the crude protein content of pollen.
3. Reliable analysis of protein‐bound and free amino acids as well as an estimation of crude protein concentration was obtained from pollen samples as low as 1 mg. Greater variation in both protein‐bound and free amino acids was found in pollen sample sizes <1 mg. Due to the variability in recovery of amino acids in smaller sample sizes, we suggest a correction factor to apply to specific sample sizes of pollen in order to estimate total crude protein content.
4. The method described in this paper will allow researchers to explore the composition of amino acids in pollen and will aid research assessing the available nutrition to pollinating animals. This method will be particularly useful in assaying the pollen of wild plants, from which it is difficult to obtain large sample weights.

## 1 INTRODUCTION

Many pollinators and other flower visitors consume floral pollen. Pollen is an important source of proteins, lipids, starch, minerals, vitamins and other nutrients in pollinator diets (Stanley & Linskens, 1974). However, the nutritional composition of pollen has been understudied with few plant species analysed so far. Poor nutrition caused by floral resource depletion from habitat loss is almost certainly a contributing factor to pollinator decline worldwide (Vanbergen, 2013). Identifying the contribution of specific plant species to the nutrition of pollinators is critical to the selection of plants to improve pollinator habitat. For this reason, methods that advance the study of pollen composition are vitally important for pollinator conservation.

The nutritional composition of pollen has most often been described in terms of total protein content when discussing the nutritional value to pollinators. Measuring protein in pollen has historically been accomplished using the Bradford assay (Bradford, 1976), or measuring nitrogen and applying a correction factor to estimate crude protein via micro‐Kjeldahl digestion or combustion methods (Buchmann, 1986; Roulston, Cane, & Buchmann, 2000). These techniques give an estimate of the percentage composition of protein, but they also require large sample sizes of raw material (1 mg–1 g depending on method). This is a recurring problem for pollination ecologists because few plant taxa produce abundant pollen that is easily collected by hand (e.g. anemophilous trees). For example, in an early study of pollen production in clover (*Trifolium repens*), only 7 mg of pollen was recovered from 373 anthers (Percival, 1950). To circumvent this, numerous researchers have used pollen pellets collected by bees (Cook, Awmack, Murray, & Williams, 2003; González‐Paramás, Bárez, Marcos, García‐Villanova, & Sánchez, 2006; Hanley, Franco, Pichon, Darvill, & Goulson, 2008; Höcherl, Siede, Illies, Gätschenberger, & Tautz, 2012; Nicolson & Human, 2013; Roulston & Cane, 2002; Somme et al., 2015; Vanderplanck, Leroy, Wathelet, Wattiez, & Michez, 2014). A problem with this approach is that bees may mix many different pollens on the pellet and as such, these pellets are often not truly monofloral.

Measurement of total protein gives an estimate of pollen's nutritional value to pollinators, but it does not reveal whether a plant's pollen contains all of the essential amino acids needed by pollinators. Acid hydrolysis has been used to quantify protein‐bound amino acids/peptides in several recent studies of pollen chemistry (González‐Paramás et al., 2006; Human & Nicolson, 2006; Nicolson & Human, 2013; Somme et al., 2015; Vanderplanck et al., 2014). Standard methods for hydrolysis of proteins to enable amino acid quantification involve acidic digestion with 6 M HCl, boiled at 110°C for 24 hr (Blackburn, 1978; Fountoulakis & Lahm, 1998) and are more efficient when they are microwaved than when they are boiled in an oven or otherwise (Marconi, Panfili, Bruschi, Vivanti, & Pizzoferrato, 1995). After hydrolysis, amino acids in the hydrolysate can then be detected and quantified using gas chromatography (GC) or high performance liquid chromatography (HPLC) techniques. Free amino acids are predominantly found on the outside of the pollen grain (pollenkitt) with lower values thought to be present within the cytoplasm (González‐Paramás et al., 2006). Free amino acids can also be measured, using HPLC after washing pollen in methanol (Cook et al., 2003), ethanol (Mondal, Parui, & Mandal, 1998) or acid (Grunfeld, Vincent, & Bagnara, 1989; but see alternate methods used by Weiner, Hilpert, Werner, Linsenmair, & Blüthgen, 2010). These methods have mainly been applied to pollen when large sample amounts were available (e.g. >3 mg pollen/sample, Bartolomeo & Maisano, 2006; Nicolson & Human, 2013; Somerville & Nicol, 2006; Vanderplanck et al., 2014).

Another recent study compared polypeptide analysis methods for protein estimation to the analysis of pollen protein hydrolysates analysed for amino acids using HPLC for 10 plant species (Vanderplanck et al., 2014). This paper also compared these methods to the more commonly used Kjeldahl method. They found that the Kjeldahl method and the hydrolysis –HPLC method yielded similar results. The total protein estimates from Kjeldahl and protein hydrolysis were sometimes twice as high as those reported by their polypeptide assay (Vanderplanck et al., 2014). These authors argue that the Kjeldahl and protein hydrolysis overestimated the total protein as a result of non‐protein sources of nitrogen. While this could be true for the Kjeldahl method that measures total nitrogen, it would not be true of the protein hydrolysis/HPLC method for amino acid analysis. Furthermore, all of the pollen analysis methods reported by Vanderplanck et al. (2014) required sample sizes of >3 mg/pollen per sample and did not use a purified protein to compare the efficiency of the methods to a known protein standard.

The pollen produced by each flower of most plant species is often much less (<1 mg) than the sample sizes used previously for analysis. No studies to date have adapted pollen analysis methods to very small pollen samples that are most likely to be collected by ecologists in the field studying plant–polliator interactions. Here, we describe a new method for the quantification of free and protein‐bound amino acids found in very small samples of pollen. We used a combination of methanol washing to collect free amino acids and acid hydrolysis to analyse protein‐bound amino acids. We identified how sample size affected the amino acids returned from both free‐amino acid washes and protein‐bound hydrolysis of samples of *Cistus* spp pollen collected by honeybees. We also developed a method for back‐calculating the total amount of protein in pollen based on the efficiency of the hydrolysis methods by comparing the hydrolysis of pollen to the hydrolysis of a known, purified protein, bovine serum albumin (BSA). With these methods, future researchers will be able to analyse small pollen sample sizes (from a much wider range of plant species) to determine free and protein‐bound amino acids and estimate total pollen protein.

## 2 MATERIALS AND METHODS

### 2.1 Optimisation of protein hydrolysis

Three experiments were conducted to optimise acid hydrolysis for small amounts of protein. Using BSA (A‐7906; Sigma‐Aldrich, St. Louis, MO, USA 98% purity), we tested: (1) whether volume of acid; (2) the amount of sample and; (3) the sample:acid ratio affected the efficiency of hydrolysis. In experiment 1, the amount of sample was constant (1 mg) but the volume of acid (HCl 6 M) varied (range: 10–400 μl HCl; *n* = 7; Table S1). In experiment 2, the amount of acid was constant (100 μl) but the amount of sample varied (range: 0.1–4 mg; *n* = 9; Table S1). In experiment 3, the volume of acid used was proportional to the amount of sample i.e. we used a fixed ratio of 1:100 (mg:μl) of sample to acid (*N* = 5).

Bovine serum albumin was weighed into 1.5 ml microcentrifuge tubes and the relative amounts of acid added (Table S1). The microcentrifuge tube lids were sealed and the samples were vortexed for 30 s. Each sample was placed in a plastic microcentrifuge box, lid secured, and the box was placed in a 900 W domestic microwave with a glass beaker containing 600 ml water (to absorb excess radiation; Zhong, Marcus, & Li, 2005) at full power for 20 min. Once finished, samples were left to cool in the microwave, then tubes were moved to a heat block and the lids were opened. The acid was evaporated at 100°C until dry. Once dry, 1 ml deionised ultra high performance liquid chromatography (UHPLC) gradient grade water was added and the tubes were mixed on a vortex for 15 min, then centrifuged at 13,249 *g* for 30 min. Supernatant was removed with a sterile 1 ml syringe (Tuberculin luer, Becton, Dickinson and Company, Franklin Lakes, NJ, USA) and passed through a 0.45 μm syringe filter (Whatman Puradisc 4 syringe filter, Maidstone, UK; 0.45 μm, nylon) to remove any remaining particulates. Filtrate was centrifuged at 13,249 *g* for a further 10 min. All samples were analysed for amino acids using UHPLC (see below).

### 2.2 Free amino acid extraction and protein hydrolysis of pollen

Commercially available honeybee‐collected pollen pellets (Kiki Ltd. Rock Rose pollen, Norfolk, UK) were used to test how efficient the free amino acid extraction and acid hydrolysis methods are on small sample sizes. Bee‐collected pollen from a monofloral crop was used in order to find a source of pollen material that was sufficient for repeated sampling (e.g. *c*. 350 mg of material). Pellets were dried at 65°C for 48 hr and lightly ground with a pestle and mortar to form a homogenate. Pollen was weighed in to 1.5 ml microcentrifuge tubes at a range of small and large sample sizes. “Small” sample sizes included 0.1, 0.2, 0.3, 0.4 (*N* = 5) and 0.5 mg (*N* = 20). “Large” sample sizes included 1, 2, 3, 4 and 5 mg (*N* = 20). In order to reduce error in sample weighing, one person repeatedly weighed all samples of pollen.

In order to extract free amino acids and to wash off any honeybee‐added sugars, 200 μl of UHPLC gradient grade methanol was added to each tube, vortexed for 1 min and then left to stand at RT (*c*. 20°C) for 10 min before being mixed again for 1 min. Tubes were centrifuged for 30 min at 13,249 *g* and the supernatant (containing the free amino acids) was removed into a clean 1.5 ml microcentrifuge tube. The remaining pollen pellet was retained for analysis of protein‐bound amino acids (see below). The methanol extract of free amino acids was dried down at 70°C in a heat block and recovered in 300 μl UHPLC gradient grade water. The extract was then passed through a 0.45 μm syringe filter to remove particulates. Once filtered, samples were stored in a freezer at −20°C until UHPLC analysis.

Amino acids were hydrolysed from proteins in the pollen pellet using the microwave‐assisted acid hydrolysis method described above. For each sample size category, we used a fixed ratio of 1:100 (mg:μl) of sample to acid. Acid was added to microcentrifuge tubes and vortexed briefly. The tubes were then hydrolysed using the microwave‐assisted method, as described above. All samples were subsequently analysed for amino acids using UHPLC.

### 2.3 UHPLC analysis of free and hydrolysed protein‐bound amino acids

UHPLC (Ultimate 3000 system; Thermo Fisher Scientific, Waltham, MA, USA) was used to measure the concentrations of 21 amino acids in all BSA and pollen samples: aspartic acid (asp), glutamic acid (glu), asparagine (asn), serine (ser), glutamine (gln), histidine (his), glycine (gly), threonine (thr), arginine (arg), alanine (ala), tyrosine (tyr), cysteine (cys), valine (val), methionine (met), gamma‐aminobutyric acid (GABA), tryptophan (trp), phenylalanine (phe), isoleucine (ile), leucine (leu), lysine (lys) and proline (pro; listed in order of elution off the column). Immediately before injection, using an automated pre‐column derivitisation programme for the autosampler (Ultimate 3000 Autosampler, Dionex, Thermo Fisher Scientific Inc.), 10 μl of sample was treated for 1 min with 15 μl of 7.5 mmol/L o‐phthaldialdehyde (OPA) and 225 mmol/L 3‐mercaptopropionic acid in 0.1 M sodium tetraborate decahydrate (Na_2_B_4_O_7_·10 H_2_O), pH 10.2 and for 1 min with 10 μl of 96.6 mmol/L 9‐fluroenylmethoxycarbonyl chloride (FMOC) in 1 M acetonitrile. This was followed by the addition of 6 μl of 1 M acetic acid. Thirty microliter of the amino acid derivatives were then injected onto a 150 × 2.1 mm Accucore RP‐MS (Thermo Fisher Scientific Inc.) uHPLC‐column. Elution of the column occurred at the constant flow rate of 500 μl/min using a linear gradient of 3%–57% (v/v) of solvent B over 14 min, followed by 100% solvent B for 2 min and a reduction to 97% solvent B for the remaining 4 min. Elution solvents were: A = 10 mmol/L di‐sodium hydrogen orthophosphate (Na_2_HPO_4_), 10 mmol/L Na_2_B_4_O_7_·10H_2_O, 0.5 mmol/L sodium azide (NaN_3_), adjusted to pH 7.8 with concentrated HCl, and B = acetonitrile/methanol/water (45/45/10 v/v/v). The derivatives were fluorometrically detected (Ultimate 3000 RS Fluorescence Detector, Dionex, Thermo Fisher Scientific, OPA: excitation at 330 nm and emission at 450 nm, FMOC: excitation at 266 nm and emission at 305 nm) and quantified by automatic integration after calibration of the system with known amino acid standards. Reference curves were obtained twice per day for all amino acids by injecting calibration standards (a pre‐made solution of 17 amino acid standards for fluorescence detection (Sigma‐Aldrich) was used. The missing four amino acids (asparagine, glutamine, GABA and tryptophan, available in solid form from Sigma‐Aldrich) were added to the solution for system calibration with mean concentrations of 25 mol/ml). Reference curve calibrations were repeated to ensure accuracy in peak identification given the normal daily variation in elution times for amino acids on the system (standard chromatogram shown in Figure S1). Elution profiles were analysed using Chromeleon (Thermo Fisher Scientific Inc.), which automatically calculates solute concentrations (nmol/ml) based on a range (different dilutions) of pre‐programmed reference curves for each amino acid based on the standards (Figure S1). Amino acid peaks were automatically detected based on pre‐calibrated elution times in the software, Chromeleon (Dionex, Thermo Fisher Scientific). All peaks were checked to ensure correct identification by the software. If amino acid peaks were wrongly assigned by the software, they were manually assigned by selecting the peak area of the correct peak, identified by retention time of the standard. Chromeleon output (micromoles per litre, μM) was converted into a standardised unit (μg/mg) in order to compare the efficiency of methods used.

### 2.4 C–H–N combustion analysis

Samples of BSA and the rock rose pollen were sent to Elemental Microanalysis Lab (http://www.elementallab.co.uk/) for CHN analysis. The CHN analysis was carried out on a CE Instruments elemental analyser model EA1110 at a combustion temperature of 1,000°C, and an exothermic tin combustion of up to 1,600°C. The technique used was high temperature combustion followed by GC separation and detection by thermal conductivity. For the GC separation, helium was used as the carrier gas at a flow rate of 120 ml/min with a 2 m packed column (Porapak QS 50/80 mesh, Elemental Microanalysis, Okehampton, UK). The GC oven temperature was an isothermal 65°C. The elemental analyser was calibrated and verified using certified reference chemicals traceable to NIST primary standards. Cyclohexanone 2,4‐dinitrophenylhydrazone and cystine were used as reference standards.

### 2.5 Statistical analysis

Statistical analyses were carried out, using spss v.23. A one‐way ANOVA was used to compare the effect of pre‐treatment on the measured protein content of pollen in the Bradford assay. Sample sizes of BSA and pollen used in hydrolysis experiments were analysed separately. Total amino acids recovered from hydrolysis of both BSA and rock rose pollen were compared in a generalised linear model, comparing source and weight of the protein. To compare the distribution of amino acids quantified in the free amino acid extraction and hydrolysis experiments, values for each amino acid were square root transformed (√(*x* + 1)) and used in a factor analysis (principal components analysis, PCA) to reduce variables into significant factors with similar correlations in relationships of amino acids. Tryptophan and GABA were removed from the dataset prior to analysis because they were present at values <0.1 ng/mg of pollen. Factors produced from the PCA were then used as dependant variables in a multivariate analysis of variance (MANOVA) using pollen weight as a main effect. The total free‐ and protein‐bound amino acids recovered from hydrolysis of pollen were compared in a MANOVA using pollen weight as a main effect and total free amino acids and total protein‐bound amino acids as separate dependant variables. For all methods specifying a normal distribution, tests for normality and homogeneity of variance were first carried out prior to analysis.

## 3 RESULTS

### 3.1 Protein hydrolysis efficiency depends on protein source and sample weight

**Figure 1:**
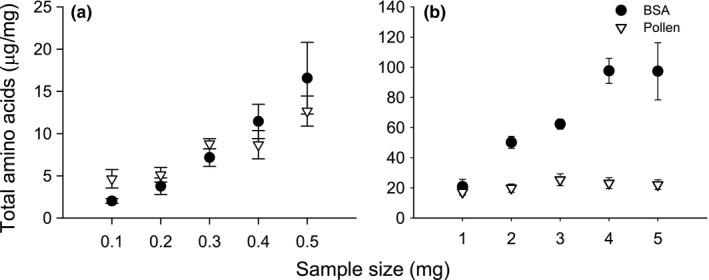
Comparison of total mean amino acids quantified from a microwave‐assisted acid hydrolysis of (a) low and (b) high weights of BSA and Rock rose pollen. Bars represent standard error of mean

The total protein‐bound amino acids (μg/mg) rendered by the hydrolysis of BSA increased with the amount of sample analysed (Figure 1, linear regression, *r* ^2^ = .842, *F*_1,42_ = 255.6, *p* < .001). A positive, but weaker relationship between sample size and protein‐bound amino acids was also observed for the hydrolysate of pollen (linear regression, *r* ^2^ = .134, *F*_1,138_ = 21.4, *p* < .001). The relative increase in amount of protein‐bound amino acids released by acid hydrolysis as a function of sample size was not significantly different between BSA and pollen for the small sample sizes (Figure 1a, GLM, protein×weight $\chi_{4}^{2}$ = 3.74, *p* = .443). In this case, as the amount of sample increased, so did the amino acids rendered by hydrolysis, as would be expected.

The relationship was different for samples ≥1 mg. In this case, the total protein bound‐amino acids rendered by hydrolysis increased as a function of the sample size for BSA but not for pollen (Figure 1b, GLM, protein×weight $\chi_{4}^{2}$ = 65.76, *p* < .001). The amount of amino acids rendered by hydrolysis of BSA plateaued for samples larger than 4 mg (LSD post hoc, *p* = .979; Figure 1b). However, we found no significant difference between the amounts of protein‐bound amino acids rendered for sample sizes of 1–5 mg of pollen (LSD post hocs, *p* > .05; Figure 1b). In a separate set of experiments, we confirmed that the “plateau” observed for the amino acids rendered by hydrolysis was due to the ratio of acid‐to‐sample (see Figure S2, Table S1), suggesting that the earlier plateau of pollen for total amino acids rendered was likely to be due to the fact that pollen had substantially less protein than BSA. We also tested whether it was necessary to first prepare the samples for hydrolysis by breaking the pollen exine wall by bead beating (Figure S3). We found that the total amino acids rendered was lower when the sample was homogenised, indicating that more sample was lost by homogenisation than when the sample was directly added to the acid (Figure S3).

### 3.2 Estimating total protein content from hydrolysed protein

For the BSA, the mean total amino acids rendered by hydrolysis ceased to change at samples ≥4 mg. The mean concentration was 97.5 μg/mg, and it was also the largest amount rendered by the hydrolysis method. If the method rendered full hydrolysis of the sample, the expected value should have been *c*. 980 μg/mg (the manufacturer specified purity of the protein was ≥98%). To verify that these samples were 98% BSA, we performed a C–H–N combustion analysis. The CHN analysis returned an average value for total nitrogen of 13.43 ± 0.03 (*N* = 3 samples). Using a 6.08 conversion factor of nitrogen to protein based on the amino acid sequence of BSA (Mariotti, Tomé, & Mirand, 2008), we estimated that the total protein in our BSA standard was 81.7%. This allowed us to calculate the expected values against the observed to estimate the efficiency of hydrolysis. We estimate hydrolysis efficiency to be 11.9% (a 8.38‐fold difference between observed and expected). To calculate the percentage protein of the sample, therefore, one would multiply 0.838 by the value for the total mean amount of amino acids rendered by hydrolysis.

For the pollen samples, we found that the total mean amount of amino acids rendered by hydrolysis ceased to change for samples ≥1 mg. The mean amount calculated across the samples ≥1 mg was 22.7 μg/mg (Figure 1). If we assume that that the efficiency of hydrolysis was the same for BSA and pollen at the point of the “plateau” (e.g. *c*. 11.9%), then we estimate the total protein concentration of honeybee‐collected pollen was *c*. 19.0% (22.7 μg/mg × 0.839). To verify this estimate, we also subjected our pollen samples to CHN analysis. The total average nitrogen was 2.96 ± 0.09 (*N* = 3 pollen samples). To estimate the percentage protein, we used the standard crude protein factor of 6.25 (Mariotti et al., 2008) because the amino acid sequence for our pollen was not available. CHN analysis estimated the total percentage protein of our samples to be 18.5% (6.25 × 2.96). Thus, if the CHN analysis is correct, our method of estimating the total protein in our pollen samples by hydrolysis was out by only 0.5%.

**Figure 2:**
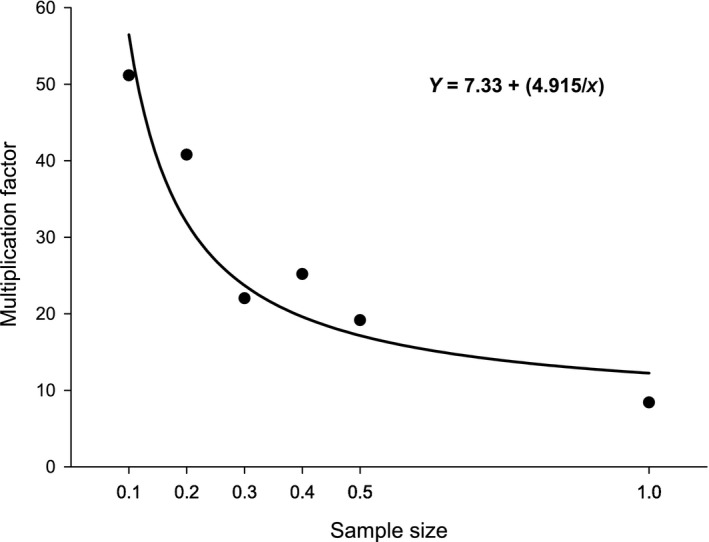
Inverse first‐order function fitted to the correction factors of low weights of pollen (0.1–0.5 mg). The correction factor for the 1 mg weight is included as a reference point for sample sizes between 0.5 and 1 mg

The efficiency of hydrolysis changed as a function of sample size for small pollen samples (<1 mg), so we were required to estimate the total protein content of the samples using a different multiplication factor. Correction factors were calculated for all lower weight samples by dividing the expected value of protein in the honeybee‐collected pollen (19.0% or 190.0 μg/mg) by the actual total amount of amino acids rendered by hydrolysis. The correction factors were regressed against sample size; the best fit to the data was a first order inverse function (Curve estimation, *r* ^2^ = 0.593, *F*_1,58_ = 84.48, *p* < .001). The equation of this line (Figure 2, *y* = 7.33 + (4.915/*x*)) was then used to calculate the specific adjustment factor for a given starting weight of pollen.

### 3.3 Sample size affects the proportions of amino acids rendered by hydrolysis

Factor analysis (principal components method) was used to test how sample size affected the proportions of free and protein‐bound amino acids recovered from pollen. Factor analysis was applied to the free and protein‐bound amino acids for the small sample sizes (0.1–0.5 mg) and large sample sizes separately (1–5 mg).

Each protein‐bound amino acid from small pollen samples was represented by one of four significant factors explaining 83.9% of the variance in the data (protein bound amino acids, Table 1, means Table S2). Pollen sample size was responsible for a change in the profile of amino acids in factors 1 and 2 but did not influence factors 3 and 4 (Table 1, MANOVA). The distribution of amino acids from the 0.5 mg samples were different to that of the 0.1–0.4 mg weights (LSD post hocs, *p* < .001), which were all similar to each other (LSD post hocs, *p* > .416).

**Table 1:** Principal components analysis (factors 1–4) and multivariate analysis of variance for protein‐bound and free amino acid distributions of low weights of pollen (0.1–0.5 mg). The amino acids represented by factors 1 and 2 are significantly different for the low sample weights. Factor loadings (in bold) indicate the amino acids with the strongest correlations for each factor

	Factors					Factors			
	1	2	3	4		1	2	3	4
Protein‐bound amino acids					Free amino acids				
Eigenvalue	7.471	3.261	2.234	1.205	Eigenvalue	5.667	4.996	1.441	1.032
Variance %	43.95	19.18	13.14	7.09	Variance %	33.34	29.39	8.48	6.07
Amino acids					Amino acids				
Ala	**0.692**	0.539	−0.292	−0.283	Ala	**0.784**	−0.043	0.051	0.363
Arg	−0.346	−0.149	**0.865**	0.215	Arg	0.09	**0.771**	−0.102	−0.217
Asp	**0.768**	−0.278	0.382	−0.131	Asp	−0.255	**0.921**	0.204	0.012
Cys	0.108	**0.779**	0.398	0.308	Cys	**0.568**	0.203	−0.336	−0.02
Glu	0.118	**0.797**	0.232	−0.021	Glu	**0.619**	−0.505	0.354	0.025
Gly	0.421	−0.177	**0.806**	0.002	Gly	0.23	0.546	−0.229	**0.592**
His	**0.668**	−0.138	0.141	−0.46	His	**0.856**	−0.449	0.089	−0.001
Ile	**0.718**	−0.146	−0.262	0.102	Ile	−0.181	0.417	**0.616**	0.465
Leu	**0.914**	0.154	0.063	0.214	Leu	0.278	**0.804**	0.301	0.078
Lys	**0.766**	0.128	−0.029	0.460	Lys	**0.796**	−0.436	0.086	−0.005
Met	**0.813**	−0.065	0.365	−0.244	Met	−0.463	**0.651**	0.207	−0.171
Phe	**0.836**	−0.101	−0.205	0.419	Phe	**0.568**	0.454	0.016	−0.258
Pro	**−0.688**	0.585	0.029	−0.184	Pro	−0.097	−0.297	**0.728**	−0.24
Ser	**−0.665**	0.614	0.042	0.276	Ser	**0.940**	0.215	−0.006	−0.011
Thr	**0.723**	0.506	0.121	−0.283	Thr	**0.765**	0.478	−0.019	−0.007
Tyr	0.482	**0.711**	−0.176	−0.105	Tyr	**0.831**	0.162	0.155	−0.280
Val	**0.816**	−0.142	−0.272	0.229	Val	0.198	**0.852**	−0.114	−0.217
Test stat *F*	24.393_4,35_	5.514_4,35_	1.018_4,35_	0.695_4,35_	Test stat *F*	3.875_4,35_	14.195_4,35_	0.763_4,35_	1.273_4,35_
*p*‐value	**<.001**	**.002**	.412	.6	*p*‐value	**.01**	**<.001**	.557	.299

Free amino acids in the small sample sizes were reduced to four significant factors, accounting for 77.9% of the variance in the data (free amino acids, Table 1, means Table S3). Sample size significantly influenced the amino acid profile in factor 1 and 2 but not in factor 3 and 4 (Table 1, MANOVA). The distribution of amino acids in 0.5 mg samples were different to all other pollen weights (Factor 2, LSD post hocs, *p* < .001).

The distribution of amino acids in pollen sample sizes of 1 mg or greater was stable and did not vary as a function of sample size (Table 2). As before, the protein‐bound amino acids were reduced to four significant factors (75.9% of variance, protein bound amino acids, Table 2, means Table S4). Sample size did not affect the amino acid profile of the protein‐bound amino acids for any of the factors (Table 2, MANOVA). This was also true for the free amino acid profiles of these samples (Table 2, MANOVA, means Table S5).

**Table 2:** Principal components analysis and multivariate analysis of variance for protein‐bound and free amino acid distributions of high weights of pollen (1–5 mg). There are no significant differences in amino acid distribution (represented by all factors) between the high sample weights. Factor loadings (in bold) indicate the amino acids with the strongest correlations for each factor

	Factors					Factors		
	1	2	3	4		1	2	3
Protein‐bound amino acids					Free amino acids			
Eigenvalue	7.343	2.718	1.772	1.381	Eigenvalue	10.184	1.495	1.082
Variance %	43.20	15.99	10.42	8.12	Variance %	59.91	8.80	6.37
Amino acids					Amino acids			
Ala	0.573	**−0.584**	0.386	0.133	Ala	**0.688**	0.14	0.489
Arg	−0.168	**0.887**	−0.114	−0.096	Arg	**0.903**	−0.053	−0.189
Asp	0.600	**0.571**	−0.001	−0.038	Asp	**0.856**	−0.376	0.058
Cys	**0.841**	0.216	0.118	−0.302	Cys	**0.936**	−0.001	−0.013
Glu	**0.561**	0.201	0.395	−0.014	Glu	0.226	**0.753**	−0.126
Gly	0.009	**0.872**	0.155	−0.105	Gly	**0.754**	−0.127	0.339
His	0.303	0.206	0.187	**0.758**	His	**0.856**	−0.225	0.08
Ile	**0.872**	−0.102	−0.39	0.027	Ile	**0.819**	−0.03	−0.125
Leu	**0.885**	0.175	−0.087	−0.016	Leu	**0.918**	0.053	−0.077
Lys	**0.778**	−0.028	−0.321	−0.006	Lys	**0.524**	0.332	−0.498
Met	**0.930**	0.094	0.061	−0.104	Met	**0.907**	0.012	−0.24
Phe	**0.883**	−0.136	−0.357	0.026	Phe	**0.804**	0.198	−0.221
Pro	−0.326	0.192	**0.744**	0.093	Pro	**0.456**	−0.432	−0.119
Ser	0.062	0.271	−0.254	**0.806**	Ser	**0.973**	0.054	0.088
Thr	**0.760**	0.182	0.379	−0.037	Thr	**0.903**	0.134	0.026
Tyr	**0.583**	−0.383	0.475	0.056	Tyr	**0.815**	−0.093	0.209
Val	**0.832**	−0.127	−0.002	0.010	Val	0.219	**0.572**	0.473
Test stat *F*	0.808_4,95_	0.219_4,95_	0.611_4,95_	1.272_4,95_	Test stat *F*	0.279_4,95_	0.456_4,95_	0.336_4,95_
*p*‐value	.523	.927	.656	.286	*p*‐value	.891	.768	.853

The same analysis was conducted for the amino acids rendered by hydrolysis of BSA for both small and large sample sizes (Table S6). The amino acid profile varied as a function of sample size for the small samples (Table S6). Sample sizes smaller than 0.4 mg did not render the same amino acid profile as the 0.4 and 0.5 mg samples (LSD post hocs, *p* < .001, means Table S7). Similarly, with larger sample sizes (varimax rotation), the amino acid distribution varied in samples lower than 4 mg (Table S6). However, all amino acids, except for aspartic acid and alanine, were similar in the 4 and 5 mg sample sizes (LSD post hocs, *p* < .001, means Table S8).

### 3.4 Correction factors

**Figure 3:**
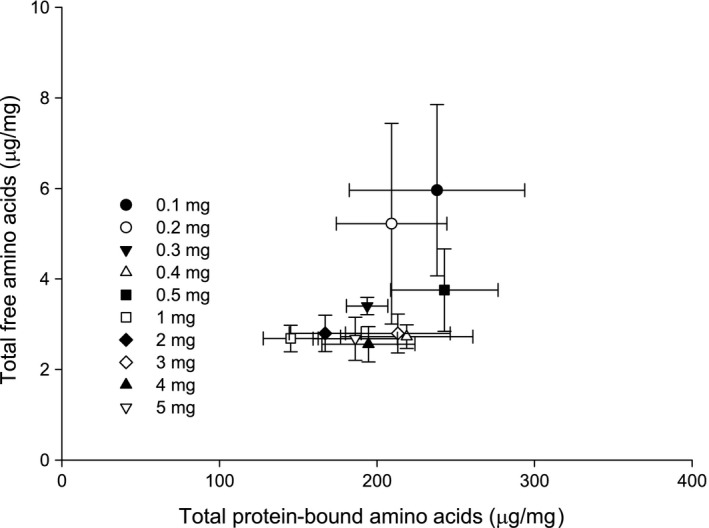
Bivariate plot of mean total protein‐bound (after correction factor) and mean total free amino acids recovered from microwave‐assisted acid hydrolysis of varying weights of pollen

Correction factors were applied to the total protein‐bound amino acids for each sample size (Table 3). The protein‐bound amino acids were then plotted against the free amino acids in a bivariate plot (Figure 3). The greatest outliers with respect to the estimation of the true values of the total free and protein bound amino acid values were for sample sizes of ≤0.2 mg. This indicates that the method of protein estimation we show is reliable for samples of >0.3 mg of pollen using this method of hydrolysis.

**Table 3:** Multiplication factors to apply to protein‐bound amino acids from each weight of pollen used in hydrolysis experiments

Pollen weight (mg)	Correction factor
0.1	51.16
0.2	40.78
0.3	22.02
0.4	25.19
0.5	19.15
1	8.39
2	8.39
3	8.39
4	8.39
5	8.39

## 4 DISCUSSION

Acid hydrolysis combined with HPLC analysis has been used to quantify protein‐bound amino acids/peptides in several recent studies of pollen chemistry (González‐Paramás et al., 2006; Human & Nicolson, 2006; Nicolson & Human, 2013; Somme et al., 2015; Vanderplanck et al., 2014). However, in all of these studies, large sample sizes of mostly bee collected pollen were used for analysis. Hand collection of pollen from floral anthers is time consuming and difficult, and in some species of plants, nearly impossible to collect in large amounts. Therefore, conventional methods that require large sample sizes limit the plant species that can be analysed in terms of pollen chemistry. Here, we describe a method for the analysis of free and protein bound amino acids in pollen rendered from small sample sizes. Though we used bee‐collected pollen to perform the study, we expect that our results generalize to hand‐collected pollen because the sugars added by bees are washed off during the methanol extraction step. The data show that pollen samples ≥0.5 mg are the most reliable, but smaller sample sizes are possible to analyse and get meaningful results. Based on our plot of the corrected values for the free and protein‐bound amino acids (Figure 3), we recommend that sample sizes no smaller than 0.3 mg are processed using our method.

Our method uses partial hydrolysis of protein (i.e. not all of the protein is hydrolysed). From this partial hydrolysis, we estimate total protein by comparing the total amino acids rendered vs. an expected value of a pure protein (BSA). Unfortunately, the manufacturer was not specific about the exact purity of our sample, and it was necessary for us to validate its purity, using the CHN combustion method. The CHN method, like all methods of protein estimation, also relies on a conversion factor (Mariotti et al., 2008). If this factor is off by a small amount, it can also affect the estimate of protein (Mariotti et al., 2008). Because BSA is a protein often used as a standard in protein assays, we were able to find a previously calculated convertion factor based on the amino acid sequence of BSA (Mariotti et al., 2008). Using a combination of methods to validate the protein content of our standard improved the conversion factor that we were able to calculate for pollen based on the total amount of amino acids rendered by hydrolysis.

Using this method, we were also able to identify the amino acid profile of pollen and compare the quantities of free and protein‐bound amino acids and provide an estimate for the total amino acid/protein concentration in a specific pollen sample. No previous studies of the protein content or the amino acid profile of pollen protein have accomplished this, in part because previous works did not use a purified protein as a standard in their analyses (e.g. Vanderplanck et al., 2014). Based on our work and several other studies of the hydrolysis of protein, we expect that the hydrolysis method and total protein estimation method should be generalizable to many kinds of proteins, including the diverse proteins found in the pollen of different plant species. For example, a recently published paper showed that the total amino acid profile of pollen identified using HCl hydrolysis‐HPLC methods was stable even when pollen samples of the same species were collected at different times of year (Roger et al., 2017). This method was used to analyse the amino acid profiles of over 25 different types of pollen protein. The type of protein should not substantially affect the efficiency of hydrolysis when we use 6 M HCl in a microwave. In fact, in the field of protein analysis, acid hydrolysis is considered one of the most reliable methods of protein estimation; it is a lot more reliable than methods that depend on spectrophotometric detection of reagents binding to protein which require that the protein is also water soluble (see Fountoulakis & Lahm, 1998). One manuscript reports that very large‐sized proteins are less efficiently hydrolysed (>22 kDa) than small proteins (Sittampalam, Ellis, Miner, Rickard, & Clodfelter, 1988) when 6 M HCl hydrolysis is used at 100°C over 24 hr. However, several studies conducted since then have shown that efficiency of hydrolysis increases with temperature (e.g. Csapá et al., 1997). Based on the work of Csapá et al. (1997), we expect that the temperatures we achieve using the microwave method are sufficient to break down even very large proteins.

We found that it was possible to increase the amount of protein hydrolysed by increasing the ratio between protein and acid during hydrolysis (from 1:100 to 1:400 [mg:μl protein:acid]). However, we chose to use a smaller volume of acid because using volumes of acid greater than 500 μl per sample considerably reduces the safety of hydrolysis by microwaving. Importantly, we confirmed that the amount of protein hydrolysed by our method did not alter the relative ratios of the protein‐bound amino acids in each sample. This indicates that further hydrolysis is not necessary to identify the ratios of protein‐bound amino acids in pollen. Our analyses also confirmed that free amino acids can be reliably quantified in samples as low as 0.5 mg, but that the samples we used (bee collected pollen) varied substantially at samples lower than this. It is possible that if pure pollen was used in this analysis, and a large number of replicates were performed, that this method would also provide a reliable estimate of free amino acids for small samples.

One of the limits of the acid hydrolysis‐HPLC method is that the amino acid composition of pollen is partially altered during protein hydrolysis because of the degradation of specific amino acids. For example, tryptophan may be completely degraded whilst asparagine and glutamine are deaminated to aspartic acid and glutamic acid, respectively (Blackburn, 1978; Salo‐väänänen & Koivistoinen, 1996). Tryptophan and glutamine were not detected in any of our hydrolysed pollen samples. Correction factors can be used to quantify lost amino acids (Fountoulakis & Lahm, 1998), e.g. nonlinear least‐squares equations (Darragh, Garrick, Moughan, & Hendriks, 1996; Robel & Crane, 1972), but sample replication is often required and general correction factors may not be entirely accurate as amino‐acids have specific rates of degradation under hydrolysis conditions (Buňka, Kříž, Veličková, Buňková, & Kráčmar, 2009; Darragh et al., 1996; Rees, 1946). Microwave‐assisted acid hydrolysis improves the speed at which hydrolysis can be performed to between 1 and 30 min compared to the standard method of at least 24 hr (Fountoulakis & Lahm, 1998). This is important because reducing hydrolysis time reduces amino acid loss from samples (Buňka et al., 2009; Simpson, Neuberger, & Liu, 1976). Some amino acids can be treated before hydrolysis to reduce loss. For example, methionine and cysteine benefit from being oxidised (but oxidisation reduces the measurable tyrosine; Bech‐Andersen, Mason, & Dhanoa, 1990) while tryptophan can be treated with an alkaline hydrolysis in a separate representative sample (Fountoulakis & Lahm, 1998). However, pollen samples are often too small to be split for separate hydrolyses and control for individual amino acid loss, so this limitation is hard to overcome using our method.

Our method of microwave‐assisted acid hydrolysis of pollen is an important tool for ecologists to study the protein and amino acid content of floral pollen. In particular, because it does not require a large sample size and because it is done using inexpensive reagents in small amounts, it allows large batches of samples to be hydrolysed in rapid succession using inexpensive conventional appliances and equipment (with the exception of the HPLC). Thus, analysis is much faster, cheaper and more comparable between samples. We successfully applied microwave‐assisted acid hydrolysis to small sample sizes of pollen. Thus, pollen, which is notoriously difficult to extract from most plants, can now be analysed for protein‐bound amino acids in small sample sizes with a standardised method for estimating total pollen protein as well, permiting ecologists to study plant species that do not produce copious amounts of pollen. This is an essential step forward for ecological research because any method which facilitates the nutritional study of pollen from a large range of plant taxa will be of significant importance to understanding pollen consumer nutrition and insect‐flower interactions.

## AUTHORS’ CONTRIBUTIONS

D.S., E.F.P., A.B. and G.A.W. designed the experiments; D.S. collected the data; D.S., G.A.W. analysed the data; D.S., E.F.P., A.B., J.B., G.A.W. wrote/edited the manuscript.

## DATA ACCESSIBILITY

The data from these experiments can be found at figshare.com, https://doi.org/10.6084/m9.figshare.5281891.v1 (Stabler, Power, Borland, Barnes, & Wright, 2017).

## References

[mee312867-bib-0001] Bartolomeo, M. P. , & Maisano, F. (2006). Validation of a reversed‐phase HPLC method for quantitative amino acid analysis. Journal of Biomolecular Techniques, 17, 131. 16741240 PMC2291777

[mee312867-bib-0002] Bech‐Andersen, S. , Mason, V. C. , & Dhanoa, M. S. (1990). Hydrolysate preparation for amino acid determinations in feed constituents: 9. Modifications to oxidation and hydrolysis conditions for streamlined procedures. Journal of Animal Physiology and Animal Nutrition, 63, 188–197.

[mee312867-bib-0003] Blackburn, S. (1978). Sample preparation and hydrolytic methods In Blackburn S. (Ed.), Amino acid determination methods and techniques (pp. 7–37). New York: M. Dekker, University of Michigan.

[mee312867-bib-0004] Bradford, M. M. (1976). A rapid and sensitive method for the quantitation of microgram quantities of protein utilizing the principle of protein‐dye binding. Analytical Biochemistrsy, 72, 248–254. 10.1016/0003-2697(76)90527-3 942051

[mee312867-bib-0005] Buchmann, S. L. (1986). Vibratile pollination in Solanum and Lycopersicon: A look at pollen chemistry In D’Arcy W. G. (Ed.), Solanaceae: Biology and systematics (pp. 237–252). New York, NY: Columbia University Press.

[mee312867-bib-0006] Buňka, F. , Kříž, O. , Veličková, A. , Buňková, L. , & Kráčmar, S. (2009). Effect of acid hydrolysis time on amino acid determination in casein and processed cheeses with different fat content. Journal of Food Composition and Analysis, 22, 224–232.

[mee312867-bib-0007] Cook, S. M. , Awmack, C. S. , Murray, D. A. , & Williams, I. H. (2003). Are honey bees’ foraging preferences affected by pollen amino acid composition? Ecological Entomology, 28, 622–627.

[mee312867-bib-0008] Csapá, J. , Csapó‐Kiss, Z. , Wágner, L. , Tálos, T. , Martin, T. G. , Folestad, S. , … Némethy, S. (1997). Hydrolysis of proteins performed at high temperatures and for short times with reduced racemization, in order to determine the enantiomers of d‐and l‐amino acids. Analytica Chimica Acta, 339, 99–107.

[mee312867-bib-0009] Darragh, A. J. , Garrick, D. J. , Moughan, P. J. , & Hendriks, W. H. (1996). Correction for amino acid loss during acid hydrolysis of a purified protein. Analytical Biochemistry, 236, 199–207. 8660495 10.1006/abio.1996.0157

[mee312867-bib-0010] Fountoulakis, M. , & Lahm, H.‐W. (1998). Hydrolysis and amino acid composition analysis of proteins. Journal of Chromatography A, 826, 109–134. 9917165 10.1016/s0021-9673(98)00721-3

[mee312867-bib-0011] González‐Paramás, A. M. , Bárez, J. A. G. , Marcos, C. C. , García‐Villanova, R. J. , & Sánchez, J. S. (2006). HPLC‐fluorimetric method for analysis of amino acids in products of the hive (honey and bee‐pollen). Food Chemistry, 95, 148–156.

[mee312867-bib-0012] Grunfeld, E. , Vincent, C. , & Bagnara, D. (1989). High‐performance liquid chromatography analysis of nectar and pollen of strawberry flowers. Journal of Agricultural and Food Chemistry, 37, 290–294.

[mee312867-bib-0013] Hanley, M. E. , Franco, M. , Pichon, S. , Darvill, B. , & Goulson, D. (2008). Breeding system, pollinator choice and variation in pollen quality in British herbaceous plants. Functional Ecology, 22, 592–598.

[mee312867-bib-0014] Höcherl, N. , Siede, R. , Illies, I. , Gätschenberger, H. , & Tautz, J. (2012). Evaluation of the nutritive value of maize for honey bees. Journal of Insect Physiology, 58, 278–285. 22172382 10.1016/j.jinsphys.2011.12.001

[mee312867-bib-0015] Human, H. , & Nicolson, S. W. (2006). Nutritional content of fresh, bee‐collected and stored pollen of *Aloe greatheadii* var. *davyana* (Asphodelaceae). Phytochemistry, 67, 1486–1492. 16808932 10.1016/j.phytochem.2006.05.023

[mee312867-bib-0102] Marconi, E. , Panfili, G. , Bruschi, L. , Vivanti, V. , & Pizzoferrato, L. (1995). Comparative study on microwave and conventional methods for protein hydrolysis in food. Amino Acids, 8, 201–208. 24186329 10.1007/BF00806493

[mee312867-bib-0016] Mariotti, F. , Tomé, D. , & Mirand, P. P. (2008). Converting nitrogen into protein—beyond 6.25 and Jones’ factors. Critical Reviews in Food Science and Nutrition, 48, 177–184. 18274971 10.1080/10408390701279749

[mee312867-bib-0017] Mondal, A. K. , Parui, S. , & Mandal, S. (1998). Analysis of the free amino acid content in pollen of nine Asteraceae species of known allergenic activity. Annals of Agricultural and Environmental Medicine, 5, 17–20. 9852488

[mee312867-bib-0018] Nicolson, S. W. , & Human, H. (2013). Chemical composition of the ‘low quality’ pollen of sunflower (*Helianthus annuus*, Asteraceae). Apidologie, 44, 144–152.

[mee312867-bib-0019] Percival, M. (1950). Pollen presentation and pollen collection. New Phytologist, 49, 40–63.

[mee312867-bib-0020] Rees, M. W. (1946). The estimation of threonine and serine in proteins. Biochemical Journal, 40, 632. 16748066 10.1042/bj0400632 PMC1270019

[mee312867-bib-0021] Robel, E. J. , & Crane, A. B. (1972). An accurate method for correcting unknown amino acid losses from protein hydrolyzates. Analytical Biochemistry, 48, 233–246. 5041403 10.1016/0003-2697(72)90186-8

[mee312867-bib-0022] Roger, N. , Moerman, R. , Carvalheiro, L. G. , Aguirre‐Guitiérrez, J. , Jacquemart, A. , Kleijn, D. , … Rasmont, P. (2017). Impact of pollen resources drift on common bumblebees in NW Europe. Global Change Biology, 23, 68–76. 27234488 10.1111/gcb.13373

[mee312867-bib-0023] Roulston, T. H. , & Cane, J. H. (2002). The effect of pollen protein concentration on body size in the sweat bee *Lasioglossum zephyrum* (Hymenoptera: Apiformes). Evolutionary Ecology, 16, 49–65.

[mee312867-bib-0024] Roulston, T. H. , Cane, J. H. , & Buchmann, S. L. (2000). What governs protein content of pollen: Pollinator preferences, pollen‐pistil interactions, or phylogeny? Ecological Monographs, 70, 617–643.

[mee312867-bib-0025] Salo‐väänänen, P. P. , & Koivistoinen, P. E. (1996). Determination of protein in foods: Comparison of net protein and crude protein (N × 6.25) values. Food Chemistry, 57, 27–31.

[mee312867-bib-0026] Simpson, R. J. , Neuberger, M. R. , & Liu, T. Y. (1976). Complete amino acid analysis of proteins from a single hydrolysate. Journal of Biological Chemistry, 251, 1936–1940. 178649

[mee312867-bib-0027] Sittampalam, G. S. , Ellis, R. M. , Miner, D. J. , Rickard, E. C. , & Clodfelter, D. K. (1988). Evaluation of amino acid analysis as reference method to quantitate highly purified proteins. Journal‐Association of Official Analytical Chemists, 71, 833–838. 3047099

[mee312867-bib-0028] Somerville, D. C. , & Nicol, H. I. (2006). Crude protein and amino acid composition of honey bee‐collected pollen pellets from south‐east Australia and a note on laboratory disparity. Animal Production Science, 46, 141–149.

[mee312867-bib-0029] Somme, L. , Vanderplanck, M. , Michez, D. , Lombaerde, I. , Moerman, R. , Wathelet, B. , … Jacquemart, A.‐L. (2015). Pollen and nectar quality drive the major and minor floral choices of bumble bees. Apidologie, 46, 92–106.

[mee312867-bib-0101] Stabler, D. , Power, E. F. , Borland, A. M. , Barnes, J. D. , & Wright, G. W. (2017). Data from: A method for analysing small samples of floral pollen for free and protein‐bound amino acids. Dryad Digital Repository, https://doi.org/10.6084/m9.figshare.5281891.v1 10.1111/2041-210X.12867 PMC5856064 29576862

[mee312867-bib-0030] Stanley, R. G. , & Linskens, H. F. (1974). Pollen: Biology biochemistry management. Heidelberg, Germany: Springer Science & Business Media.

[mee312867-bib-0031] Vanbergen, A. J. (2013). Threats to an ecosystem service: Pressures on pollinators. Frontiers in Ecology and the Environment, 11, 251–259.

[mee312867-bib-0032] Vanderplanck, M. , Leroy, B. , Wathelet, B. , Wattiez, R. , & Michez, D. (2014). Standardized protocol to evaluate pollen polypeptides as bee food source. Apidologie, 45, 192–204.

[mee312867-bib-0033] Weiner, C. N. , Hilpert, A. , Werner, M. , Linsenmair, K. E. , & Blüthgen, N. (2010). Pollen amino acids and flower specialisation in solitary bees. Apidologie, 41, 476–487.

[mee312867-bib-0034] Zhong, H. , Marcus, S. L. , & Li, L. (2005). Microwave‐assisted acid hydrolysis of proteins combined with liquid chromatography MALDI MS/MS for protein identification. Journal of the American Society for Mass Spectrometry, 16, 471–481. 15792716 10.1016/j.jasms.2004.12.017

