# Supplementary material for: A method for analysing small samples of floral pollen for free and protein‐bound amino acids
Source: Methods Ecol Evol. 2017 Oct 16;9(2):430–8. doi: 10.1111/2041-210X.12867 (PMC5856064; doi:10.1111/2041-210X.12867)
Supplement: Supplementary file 1 [file MEE3-9-430-s001.docx]

Supporting information, Stabler et al.

Figure S1. Example chromatogram showing retention times for 21 amino acids quantified. Note that GABA elutes as two peaks

Figure S2. Table S1. Total recovered amino acids from hydrolysis experiments of BSA with protein weight maintained and acid volume varied (A) and protein weight varied and acid volume maintained (B). Table to the right of the figure explains each treatment and the observed (total recovered) and expected total amino acids recovered from each treatment.

Figure S3. Total measured protein estimated using Bradford assay (A) and total measured amino acids from microwave-assisted acid hydrolysis following pre-treatments to 1 mg pollen samples.

Table S2. Mean protein-bound essential and non-essential amino acids measured from low sample sizes of pollen (0.1 – 0.5 mg) after microwave-assisted acid hydrolysis

Table S3. Mean free essential and non-essential amino acids measured from low sample sizes of pollen (0.1 – 0.5 mg) after microwave-assisted acid hydrolysis

Table S4. Mean protein-bound essential and non-essential amino acids measured from high sample sizes of pollen (1 – 5 mg) after microwave-assisted acid hydrolysis

Table S5. Mean free essential and non-essential amino acids measured from high sample sizes of pollen (1 – 5 mg) after microwave-assisted acid hydrolysis

Table S7. Mean essential and non-essential amino acids measured from low sample sizes of BSA (0.1 – 0.5 mg) after microwave-assisted acid hydrolysis

Table S8. Mean essential and non-essential amino acids measured from high sample sizes of BSA (1 – 5 mg) after microwave-assisted acid hydrolysis
