# Supplementary material for: A method for analysing small samples of floral pollen for free and protein‐bound amino acids
Source: Methods Ecol Evol. 2017 Oct 16;9(2):430–8. doi: 10.1111/2041-210X.12867 (PMC5856064; doi:10.1111/2041-210X.12867)
Supplement: Supplementary file 2 [file MEE3-9-430-s002.pdf]

Low weights BSA

	Factors	
	1	2
Eigenvalue	12.19	1.47
Variance %	71.73	8.66
Amino acids		
Ala	0.971	0.006
Arg	0.856	-0.242
Asp	0.945	0.111
Cys	0.974	0.126
Glu	0.795	0.073
Gly	0.940	0.001
His	0.878	-0.265
Ile	0.150	0.678
Leu	0.986	0.007
Lys	0.794	0.023
Met	0.748	-0.320
Phe	0.981	0.035
Pro	0.828	-0.125
Ser	0.970	0.073
Thr	0.966	0.094
Tyr	0.099	0.840
Val	0.819	0.102
Test stat F	$11.93_{4,20}$	$3.53_{4,20}$
P value	**<0.001**	**0.025**

High weights BSA

	Factors		
	1	2	3
Eigenvalue	10.11	2.61	2.38
Variance %	59.47	15.35	14.00
Amino acids			
Ala	0.137	-0.095	0.902
Arg	0.910	0.043	-0.319
Asp	0.453	0.061	0.827
Cys	0.931	-0.119	0.280
Glu	0.361	0.470	-0.247
Gly	0.942	-0.081	0.244
His	-0.002	-0.953	-0.138
Ile	0.782	0.518	0.229
Leu	0.934	-0.161	0.282
Lys	0.879	0.321	0.279
Met	0.868	0.196	-0.108
Phe	0.943	-0.184	0.190
Pro	0.120	-0.818	0.127
Ser	0.958	0.050	0.241
Thr	0.941	0.196	0.256
Tyr	0.793	0.480	0.309
Val	0.910	0.187	0.265
Test stat F	$9.91_{4,20}$	$41.32_{4,20}$	$21.53_{4,20}$
P value	**<0.001**	**<0.001**	**<0.001**
