# Supplementary material for: A method for analysing small samples of floral pollen for free and protein‐bound amino acids
Source: Methods Ecol Evol. 2017 Oct 16;9(2):430–8. doi: 10.1111/2041-210X.12867 (PMC5856064; doi:10.1111/2041-210X.12867)
Supplement: Supplementary file 3 [file MEE3-9-430-s003.docx]

Table S6. PCA and MANOVA amino acid distributions of low (0.1 - 0.5 mg) and high (1 – 5 mg) weights of BSA
